# Biphasic SMN condensates safeguard stress-induced cytoplasmic mislocalization of splicing RNPs

**DOI:** 10.1007/s00018-026-06459-9

**Published:** 2026-09-30

**Authors:** Yannick L. Riedel, Jessica Dressler, Maximilian T. Schilling, Oliver J. Gruss

**Affiliations:** Institute of Genetics, Rheinische Friedrich-Wilhelms Universität, 53115 Bonn, NRW Germany

**Keywords:** Survival motor neurons (SMN), Spinal muscular atrophy (SMA), UsnRNP, Stress, Microtubules

## Abstract

Biogenesis of UsnRNPs occurs in distinct steps in the nucleus and the cytoplasm. Sequential cytoplasmic actions of CLNS1A in the PRMT5 complex and of the SMN complex assemble the Sm core structure consisting of RNA and proteins. Nuclear SMN, condensed in Cajal Bodies, promotes late maturation steps. Whether cytoplasmic SMN undergoes condensation, and how this contributes to UsnRNP biogenesis or homeostasis, is poorly defined. Here, we show that molecular crowding stress induces rapid, reversible condensation of cytoplasmic SMN into droplets and filamentous assemblies, S-bodies, that sequester mislocalized cytoplasmic UsnRNPs along microtubules. During stress recovery, S-bodies undergo microtubule-dependent reorganization into split SMN–CLNS1A condensates, Janus bodies, that promote clearance of mislocalized UsnRNPs. Strikingly, cellular models of the SMN-associated disease spinal muscular atrophy fail to assemble S-bodies and to clear cytoplasmic UsnRNPs. Our findings identify stress-induced SMN condensation as a mechanism to buffer and resolve cytoplasmic UsnRNP mislocalization and reveal impaired tolerance to molecular crowding as a hallmark of spinal muscular atrophy.

**Supplementary Information:**

The online version contains supplementary material available at https://doi.org/10.1007/s00018-026-06459-9.

## Introduction

Spliceosomal U-rich small nuclear ribonucleoproteins (UsnRNPs) are essential for pre-mRNA splicing and require stepwise, coordinated assembly. Central to this process is the Survival Motor Neuron (SMN) complex, which promotes early assembly steps of UsnRNPs in the cytoplasm and supports late maturation steps in the nucleus. In humans, the cytoplasmic SMN complex consists of 9 subunits, the name giving SMN protein, Gemins2 to 8 and the unr-interacting protein, UNRIP. The cytoplasmic SMN complex aids formation of 7 Sm proteins around the UsnRNA, resulting in Sm core assembly. Before assembly into Sm cores, Sm proteins are stably held in kinetically trapped subcomplexes by the assembly chaperone CLNS1A (pICln) that is part of the PRMT5/methylosome complex. The SMN complex takes over Sm proteins from these intermediates in an UsnRNA-dependent manner to form Sm cores. Final cytoplasmic maturation steps include UsnRNA 5’cap hypermethylation and 3’end processing [1–6] after which the maturing UsnRNPs are targeted into the nucleus for final maturation comprising UsnRNA modifications and additional, individual protein association to UsnRNPs [7–9]. In cells producing high content of UsnRNPs, maturing UsnRNPs and the nuclear SMN complex undergo liquid–liquid phase separation and condense together with several other proteins and RNPs in Cajal bodies (CB) to supposedly support nuclear SMN functions [10–14].

Loss of SMN protein function causes spinal muscular atrophy (SMA) urging the question about the causal relationship between SMA etiology and cellular SMN functions [14–16]. In contrast to recognizing the fundamental importance of SMN in UsnRNP biogenesis, it remains less well characterized how the SMN complex responds to acute cellular stress and whether such responses are required to preserve UsnRNP homeostasis [17–19]. This question is particularly important because environmental and mechanical stresses profoundly alter molecular crowding and ribonucleoprotein organization, thereby challenging the integrity of macromolecular assembly pathways. While nuclear SMN condensation in CB is well characterized in cells with high splicing demand, the functional significance of cytoplasmic SMN condensation remains less well understood. Cytoplasmic SMN has been observed in stress-induced assemblies, including U-bodies [20–22] and stress granules [23, 24], suggesting that adaptive condensation may contribute to RNA homeostasis under adverse conditions [25]. This possibility is especially intriguing as SMA-associated mutations can also disrupt the YG box of SMN’s C-terminus, a region implicated in oligomerization and phase separation [26, 27].

Here, we identify a rapid and reversible stress-responsive condensation pathway of cytoplasmic SMN triggered by osmotic and mechanical stress. We show that SMN assembles along microtubules (MT) into distinct condensates that sequester UsnRNPs mislocalized to the cytoplasm under stress conditions and subsequently reorganize in an MT-dependent manner during recovery to promote their clearance. These condensates are distinct from canonical stress granules (SG) and undergo MT-dependent remodeling into adjacent SMN–CLNS1A assemblies that facilitate UsnRNP homeostasis. Strikingly, SMA model cells fail to mount this adaptive condensation response, resulting in defective cytoplasmic UsnRNP clearance, whereas treatment with Risdiplam restores condensate formation and recovery capacity.

Together, our findings uncover a stress-buffering function of cytoplasmic SMN condensates and identify impaired adaptation to molecular crowding stress as a previously unrecognized feature in SMA pathology.

## Materials and methods

### Reagents

**Table:** 

Cell lines	Source
IMR90 wt	ATCC_CCL-186
IMR90_eYFP_SMN_wt	Gruss Lab [28]
IMR90_eYFP_SMN_ΔEx7	Gruss Lab [28]
MEF wt	Witke Lab, Institute of Genetics, University of Bonn
MEF SMA	Gruss Lab [28]

Antibodies	Source
α-SMN, mouse monoclonal (7B10)	provided by Fischer Lab, Univ. of Würzburg
α-SMN, rabbit polyclonal	provided by Fischer Lab, Univ. of Würzburg
α-Gemin2, mouse monoclonal	SantaCruz Biotechnology SC-32896
α-Gemin3 (DDX20), mouse monoclonal	Wolfson Centre for Inherited Neuromuscular Disease (CIND)
α-methylated Sm proteins, mouse monoclonal (Y12)	Provided by Joan Steitz, Yale University
α-smB, mouse monoclonal	SantaCruz Biotechnology SC-374009
α-TMG, mouse monoclonal	Cal Biochem (NA02)
α-pICln, mouse monoclonal	SantaCruz Biotechnology SC-271327
α-Tubulin, mouse monoclonal	SantaCruz Biotechnology SC-32293

Chemicals	Source	Final concentration used
Penicillin – Streptomycin	PAN-Biotech	1%
Geniticindisulfat solution (G418)	Carl Roth Art.No. 2039.3	500 µg/mL
Saccharose	CarlRoth	400 mM
Dithiothreitol (DTT)	Biomol	2 mM
Sodium arsenite	Merck	500 µM
Nocodazole	Sigma Aldrich	10 µm
DMSO	CarlRoth	10%
Cycloheximide	Cayman Chemical	10 µM
NVP2	Cayman Chemical	10 µM
Risdiplam	Cayman Chemical	1 µM

Nucleic acids	Source
In situ sensor poly A (mRNA)	Eurofins genomics 5 ‘- [Cy 3]TTTTTTTTTTTTTTTTTTTTTTTTTTTTTT −3 ‘
In situ sensor U1 snRNA	Eurofins genomics 5 ‘- [Cy3]GTATCTCCCCTGCCAGGTAAGTAT −3
siRNA On-TargetPlus SmartPool CLNS1A	Dharmacon; L-012571–00–0005

### Cell culture conditions and treatments

#### Regular protocol

Cells were cultured in MEM standard media (Pan Biotech) containing 10% FBS, as well as 1% penicillin–streptomycin. Cells were kept at 37 °C and 5% CO_2_. IMR cells expressing an eYFP-SMN variant were cultured with additional 500 µg/mL G418 (Roth). All cells were cultured for a maximum of 15 passages. Mouse Embryonic Fibroblasts (MEF) wt and MEF SMA [28] cells were immortalized, transducing them for 24 h with viral particles, containing the SV40 Large T-Antigen. Cells were washed 24 h post transduction and kept for another 24 h in standard MEM before being passaged and taken into standard cell culture.

#### Osmotic stress and recovery

For all experiments applying osmotic stress, a 400 mM sucrose solution in MEM was prepared freshly on the day of treatment. For all experiments with an additional recovery phase, cells were washed once with MEM before being left in fresh MEM for the indicated time points.

#### Modulation of osmotic stress

For all treatments combining sucrose and inhibitors (Nocodazole, Cycloheximide, NVP2), cells were pre-treated for 1 h with the respective inhibitor before being stressed. For all time points, inhibitors were kept throughout the entire time course. Risdiplam was prepared in MEM and cells were pre-treated for 24 h prior fixation. For treatments combining Risdiplam and sucrose, Risdiplam was kept throughout the entire time course. For results in Fig. S1, chemicals were prepared in MEM standard media at the concentration shown in the list of chemicals above. Cells were treated for 3 h, respectively, before being fixed and stained with antibodies against SMN and G3BP1. For treatment with DMSO, MEM with or without sucrose was supplied with 10% DMSO. Cells were treated with DMSO containing media for 10 min prior to fixation.

#### Mechanical stress

Stably expressing eYFP-SMN_wt cells were seeded into a 35 mm glass bottom dish (Ibidi), two days prior to imaging. On the day of imaging cells were covered with low-melting agarose. A magnet was placed in a second dish and placed directly onto the agarose cushion. A second magnet was placed below the dish and the setup was placed into a Keyence BZ800 system. Images were immediately acquired with 10 s iterations with a total of 180 s of recording.

#### siRNA Knockdown

Knockdown of CLNS1A was performed using On-TargetPlus SmartPool CLNS1A siRNA (Dharmacon; L-012571–00–0005) for 72 h. siRNA transfection was performed using Lipofectamine RNAiMAX according to the manufacturer’s protocol. Please note that knockdown is not compatible with osmotic stress.

### Western blotting

Protein samples were run on a SDS-PAGE Gel and transferred onto a nitrocellulose membrane using 1 × blotting buffer containing 10% methanol. The membrane was blocked using Roti-Block (Roth) for 1 h at RT, incubated in primary antibody overnight at 4 °C and washed three times with 1 × TBS-Tween before incubation in HRP secondary antibody for 1 h at RT. The membrane was washed thrice with 1 × TBS-Tween before developing.

### Microscopy techniques

#### Fixation/sample preparation

For all standard immunofluorescence images, cells were grown on 12 mm glass cover slips (VWR) and fixed using a PHEM fixation solution for 20 min at 37 °C (PHEM buffer, 4% PFA, 0.1% Glutaraldehyde, 1% Triton X100). Commercial Roti-Immunoblock (Roth) was used to block cells prior to immuno-staining.

#### Indirect immunofluorescence

Cells were stained in a primary antibody solution for at least 1 h at RT in Roti-Immunoblock. Secondary antibodies were prepared together with DAPI in 1 × PBS. Cells were washed twice using PBS before being incubated for an additional 45 min in the secondary antibodies. Cover slips were washed again twice using 1 × PBS, as well as once in ddH_2_O before being mounted on cover slides (VWR) using Fluormount-G mounting media (Southernbiotech, Cambridge).

#### In situ hybridisation

Fixed and blocked cells were equilibrated with 2 × SSC for 5 min at RT before incubation in a wet chamber with the probe diluted 1:1000 in hybridization buffer at 37 °C for 2 h. Subsequent washing in 4× SSC and 2 × SSC allowed additional antibody staining if needed. Otherwise, cover slips were incubated for 5 min in DAPI, washed in 1 × PBS and double distilled (dd) H_2_O and mounted onto cover slides as described before.

#### Widefield imaging

Imaging for Figs. 6 and S1 was performed using a Zeiss AXIOPHOT equipped with a 100 × 1.3 NA oil immersion objective. Imaging for all remaining figures was performed on a NIKON Eclipse Ti2 system. Images were acquired using a 100 × 1.4 NA immersion oil objective. Images were processed using ImageJ version v1.54p.

#### Super resolution imaging

For Stochastical Optical Reconstruction (STORM) microscopy, cells were grown in 6-well plates on 25 mm x 25 mm high precision cover slips (VWR) and fixed using a PHEM fixation solution for 20 min at 37 °C. Commercial Roti-Immunoblock (Roth) was used to block cells prior to immuno-staining. All primary as well as secondary antibodies were prepared in Roti-Immunoblock containing 0.05% Triton X-100 in a dilution of 1:200. Primary antibodies were incubated for at least 2 h at RT.

Cells were washed twice using Dulbecco’s PBS (DPBS, Pan Biotech) before being incubated in secondary antibodies for 1 h at RT. For all STORM stainings, secondary antibodies coupled to either Alexa555 or Alexa647 were used. After secondary antibody incubation, cells were placed back into a 6-well plate and washed thrice in DPBS (Pan Biotech) for 5 min at RT on a rocker, followed by 15 min post fixation using 4% PFA. PFA was aspirated and cells washed again twice for 5 min using DPBS. Cells could now be stored at 4 °C in DPBS or used for imaging. Mounting medium/imaging buffer and Glycerol containing 5% Tris (pH 8) were prepared freshly. Vectashield (Vector Laboratories) was supplied at a final concentration of 30%. 100 µl of Vectashield-glycerol mixture was added into indented cover slides (Marienfeld, 1320000). Cover slips were taken from the 6-well and dipped thrice in ddH_2_O before being placed on the cover slide. Cover slips were mounted on the cover slides using black sticky aluminium foil with a cut-out to allow imaging of the cover slip. Imaging was performed on a NIKON Eclipse Ti2 system, using the STORM application, provided in the Nikon NIS Elements software. Images were acquired using a 100 × 1.4 NA immersion oil objective. For the STORM image in Fig. 3, the acquired image was processed using the ANNA-PALM network in ImageJ [29].

#### Live cell imaging

For live cell imaging, stably expressing eYFP-SMN_wt cells were seeded into an 8-well cell culture dish (Ibidi), two days prior to imaging. On the day of imaging, the media was replaced by MEM without phenol red. A Keyence BZ800 system equipped with an incubation chamber was used for imaging cells. Prior to sucrose exposure, an image was taken using a 60 × 1.3 NA oil immersion objective. Media was manually aspirated and replaced by MEM without phenol red, containing 400 mM sucrose. At each respective time point, cells were refocussed and manually imaged. At 180 min, the sucrose media was aspirated and replaced with sucrose-free MEM without phenol red. The same procedure was repeated once again, to wash out the remaining sucrose. During recovery, cells were again refocused and imaged manually at each indicated time point.

### Statistical evaluation of image- and sample data

#### ComDet quantification of SMN and G3BP condensates

The widefield application in the NIS Elements software was used to generate stitched images of 8 × 8 images. For imaging, a 1.5 × magnification lens on the NIKON Eclipse Ti2 system, together with a 40 × 1.4 NA oil immersion objective was used.

Images were analysed using the ComDet plug-in for ImageJ. Individual thresholds, as well as circularity and size of condensates were set for the 180 min time-point. All images were analysed according to these settings. The graph shows the average condensate number per cell (yellow curve for SMN, magenta curve for G3BP1) and the percentage of overall G3BP1 condensates colocalizing with SMN condensates (grey curve).

#### Condensate roundness quantification

Condensate roundness of the SMN signal in single S-bodies and Janus bodies was quantified using the particle analyzer in ImageJ. Therefore, a binary mask was created for each individual condensate followed by the particle analyzer calculating roundness values.

#### Cell counting

For all quantifications a sample size of minimum 100 cells were analysed in each replicate, if not indicated otherwise. For each of three replicates, mean values were calculated and plotted as individual data points. Overall mean value and -/+ standard deviation (S.D.) are shown in the graphs.

## Results

### Cytoplasmic SMN rapidly condenses upon molecular crowding stress

**Fig. 1:**
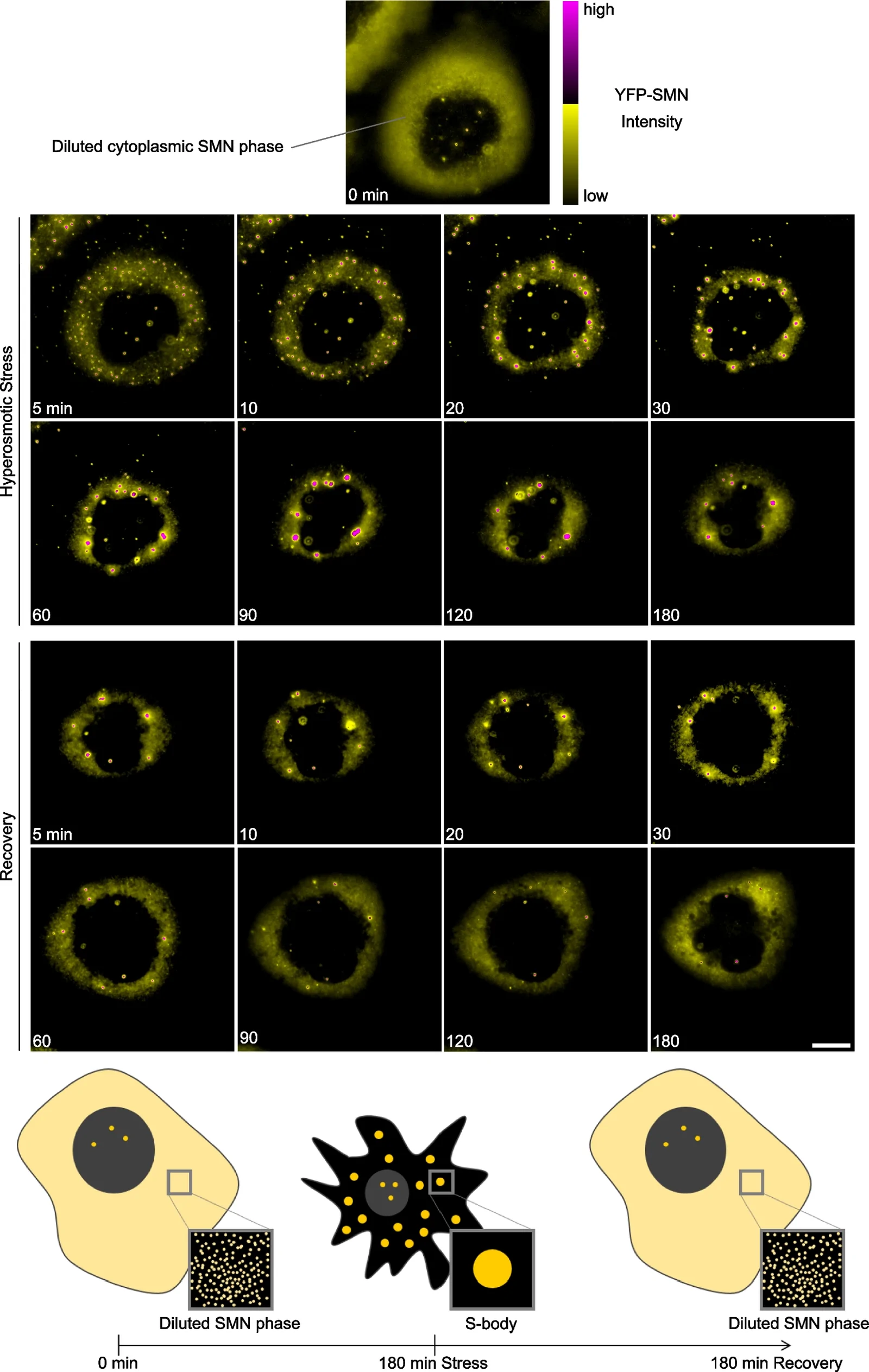
SMN forms reversible cytoplasmic condensates upon hyperosmotic stress. Still images of live IMR90 cells stably expressing YFP-SMN, which were put under osmotic stress for 180 min and recovered under non-stressed conditions for another 180 min. Images were analysed using bleach correction to enable direct intensity comparison over time. Scalebar: 10 µm. The graphic sketches SMN condensation states during the time course

Cells cope with rapid changes in molecular crowding during which macromolecular assemblies condense into liquid protein droplets [30, 31]. To analyse how the cytoplasmic SMN complex reacts to crowding stress in real time, we challenged human IMR90 lung fibroblasts stably expressing eYFP-SMN [28] with hyperosmotic stress using sucrose [30]. Live imaging revealed a rapidly unmixing eYFP signal, starting instantly, with numerous droplet-like structures appearing in the cytoplasm already 5 min after hyperosmotic stress induction (Fig. 1, Video 1). Likewise, mechanical stress led to instant unmixing of the cytoplasmic eYFP signal (Video 2). Small droplets became fewer with time, fusing to larger condensates (Fig. 1, see 20 min and later). When hyperosmotic conditions were reverted to isosmotic conditions (stress recovery), large cytoplasmic SMN condensates dissolved gradually, but in most cells completely within 180 min suggesting a kinetically asymmetric but reversible process of condensation and de-condensation.

Taken together, this shows that SMN rapidly but reversibly condenses in the cytosol in response to molecular crowding stress into condensates that we refer to as S-bodies.

### S-bodies are distinct from stress granules and U-bodies

**Fig. 2:**
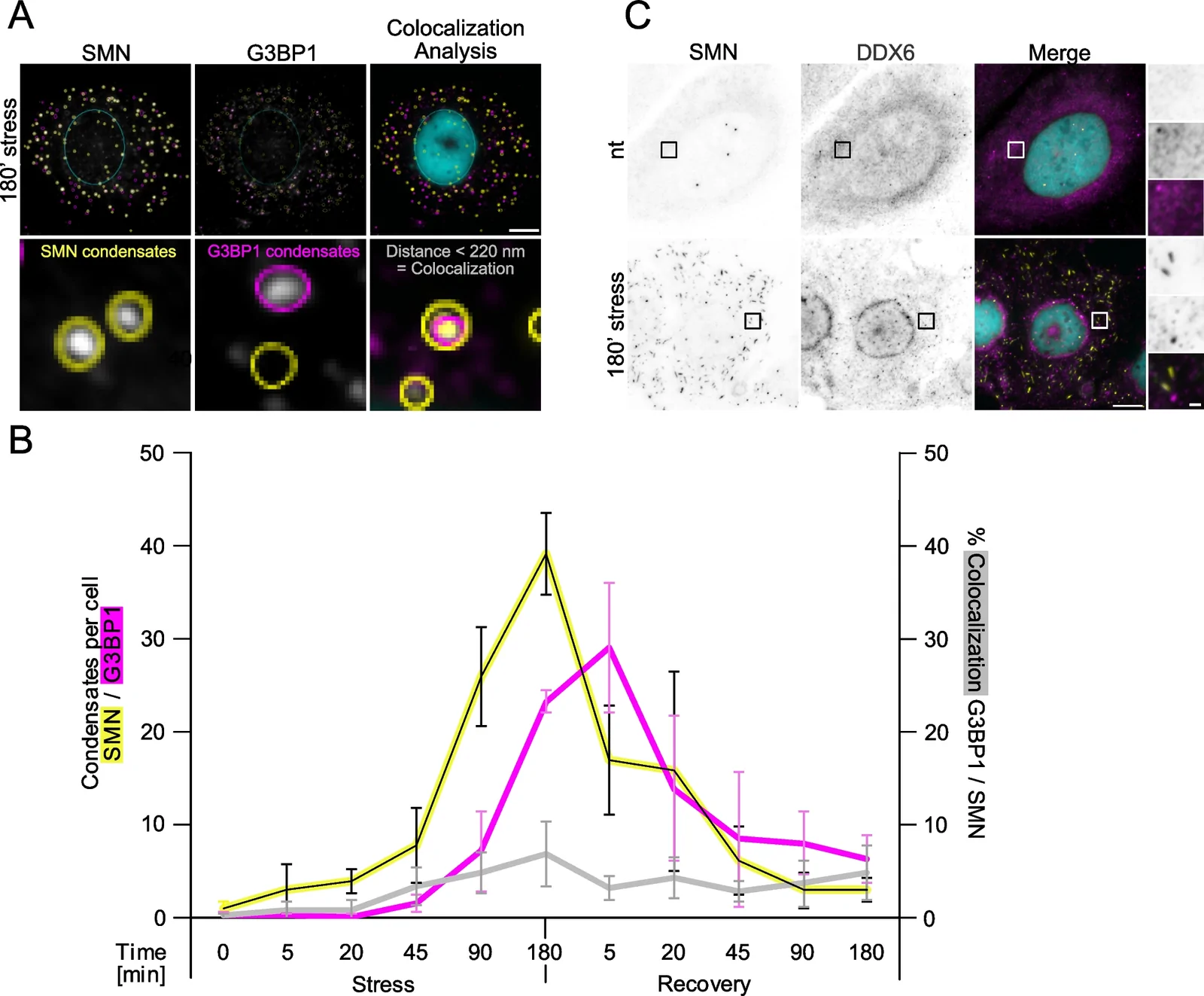
S-bodies and SG assemble separately upon osmotic stress. IMR90 cells were put under osmotic stress for 180 min (**A**, **C**) and recovered under non-stressed conditions for another 180 min (**B**), fixed and stained for endogenous SMN, the SG marker G3BP1 and the P-body marker DDX6 (**C**). B: Automated quantification (see methods) of S-body (SMN, yellow) and SG (G3BP1, magenta) condensates per cell as well as their colocalization (grey) included at least 100 cells in each of three independent experiments. The graph shows mean values and -/+ S.D

Next, we tested a potential link of the rapidly forming cytoplasmic S-bodies and stress granules (SG), which had previously been associated with SMN [23] and form e.g. after application of redox stress [32]. Cytoplasmic condensates of G3BP1 were seen as typically irregular SG-condensates after application of Arsenite or reductive (DTT) stress, while they did not accumulate endogenous SMN visualized by indirect immunofluorescence (Fig. S1). In contrast, osmotic stress led to smaller, separate condensates of G3BP1 as well as SMN (Fig. S1). To further address if S-bodies and SG assemble independently of each other, we quantified the kinetics of S-bodies and G3BP1-positive structures by indirect immunofluorescence during osmotic stress and recovery (Fig. 2A and B). The onset of condensation of SMN into S-bodies preceded the appearance of hyper-osmotic G3BP1 structures. Likewise, while cells with S-bodies dissolved them readily upon stress recovery, cells with G3BP1-positive condensates reacted with delay (Fig. 2A and B). Even though assembly and disassembly of both types of condensates occurred with similar kinetics (Fig. 2B), they did not co-localize (Fig. 2A and B). Likewise, DDX6, an established marker for Processing (P)-bodies, was not accumulated into S-bodies nor formed structures next to S-bodies (Fig. 2C). DDX6 in P-bodies has been described to consistently localize next to Uridin-rich (U)-bodies, which assemble in the cytoplasm upon viral infection and include SMN as well as cytoplasmic UsnRNPs [25].

Taken together, this data shows that S-bodies are distinct from SG and suggest that they differ from P-bodies and U-bodies. Even though SG-like structures form under osmotic stress, they assemble separately from S-bodies.

### S-bodies are microtubule associated biomolecular condensates

**Fig. 3:**
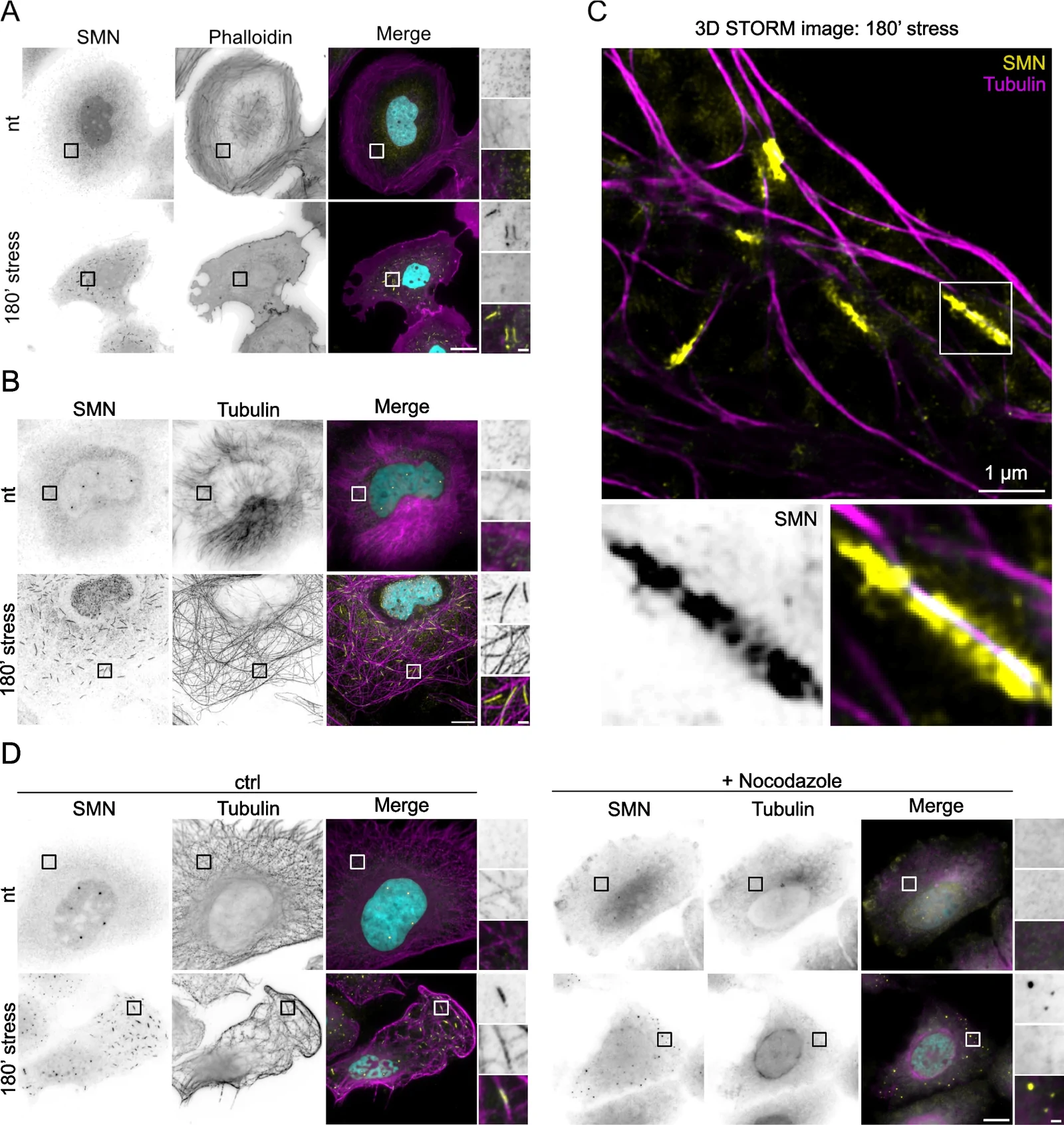
S-bodies are biomolecular condensates that bind to microtubules. IMR90 cells were fixed and analysed using antibodies against SMN (yellow in merges) and (**A**) fluorescently labelled Phalloidin (magenta) to detect F-actin or (**B**) antibodies against Tubulin (magenta) to detect MT. Scalebar: 10 µm; in insets 1 µm. (**C**) STORM image of S-bodies along MT in IMR90 cells during 180’ stress. (**D**) IMR90 cells were pretreated with nocodazole 60 min prior to and during osmotic stress (180’) and SMN and MT visualized as in (**B**). Scalebar: 10 µm; in insets 1 µm

Typical nuclear condensates of SMN in CB can be dissolved using a short DMSO treatment, consistent with liquid–liquid phase separation being the driving force for SMN condensation. Likewise, S-bodies of endogenous SMN visualized by indirect immunofluorescence appeared in droplet-like or even needle-like structures (Fig. 3), both of which were dissolved by DMSO-treatment (Fig. S2). The needle-like appearance of S-bodies indicated wetting along elongated structures, e.g. cytoplasmic filaments (Fig. 3). SMN had been described to colocalize with F-actin [33]. We therefore tested the alignment of needle-shaped S-bodies along actin filaments. However, osmotic stress generally disturbed the F-actin cytoskeleton and revealed no colocalization with S-bodies (Fig. 3A). In contrast, we detected consistent alignment of elongated S-bodies along MT (Fig. 3B). 3D Stochastical optical reconstruction microscopy to visualizeendogenous SMN confirmed that needle-shaped S-bodies aligned along MT (Fig. 3C). Consistent with a MT-dependent shaping principle, needle-like S-bodies disappeared when MT were depolymerized using Nocodazole (Fig. 3D).

This shows that S-bodies behave as biomolecular condensates and bind to MT. MT association likely enables reshaping of S-bodies into needle-like structures.

### S-bodies cover mislocalized UsnRNPs during stress

**Fig. 4:**
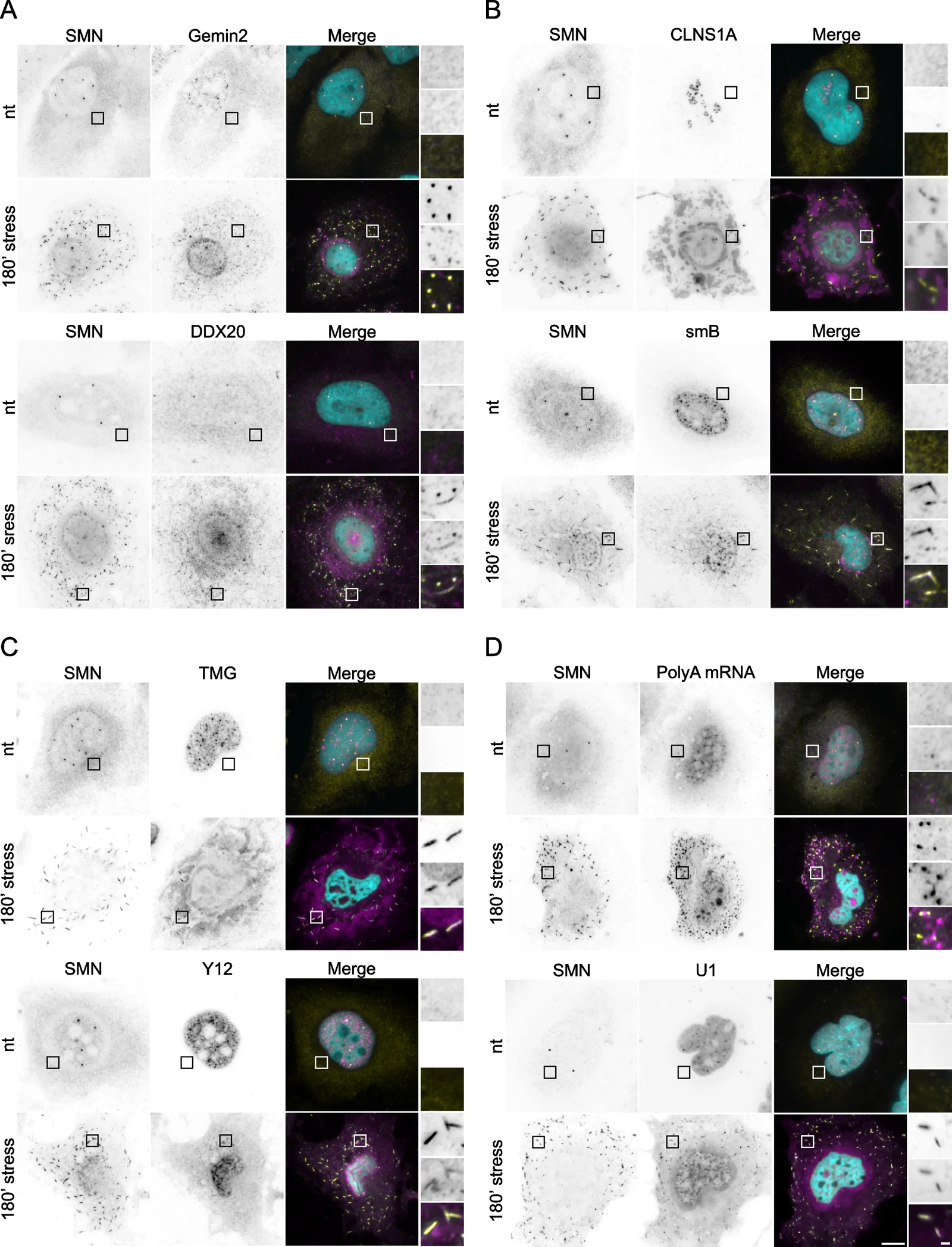
S-bodies comprise the SMN complex and mislocalized cytoplasmic UsnRNPs. IMR90 cells were put under osmotic stress for 180 min and SMN visualized via indirect immunofluorescence (yellow in merges) together (magenta in merges) with (**A**) Gemin 2 and DDX20, (**B**) CLNS1A/pICln and Sm B, (**C**) methylated Sm proteins (Y12 antibody) and the hypermethylated UsnRNA CAP structures (TMG antibody), (**D**) mRNAs (in situ hybridization using fluorescently labelled oligo dT) and U1 snRNA (in situ hybridization using fluorescently labelled U1 specific probe). Scalebar: 10 µm; in insets 1 µm

We next tested if S-bodies incorporated SMN complex proteins beyond SMN itself. Therefore, hyperosmotic-challenged IMR90 cells were stained with antibodies against SMN and Gemin2 or DDX20 (Gemin3). Both Gemins colocalized with SMN in CB and S-bodies (Fig. 4A). Furthermore, we also detected Sm proteins, exemplified by SmB, to co-condense in S-bodies. In contrast, SMN’s partner chaperone for Sm proteins, CLNS1A, did not accumulate in S-bodies upon hyperosmotic stress (Fig. 4B).

The appearance of readily visible cytoplasmic SMN and Sm protein-containing condensates upon stress posed the question if these were intermediates of ongoing UsnRNP assembly or UsnRNPs mislocalized from the nucleus to the cytoplasm. To dissect these possibilities, we performed further colocalization studies. The Y12 antibody, which mostly detects Sm proteins of mature UsnRNPs, and one that detects the hypermethylated CAP-structure (2,2,7, trimethylguanosine, TMG) of mature UsnRNPs, both decorated S-bodies (Fig. 4C). Consistently, fluorescence in situ hybridization revealed U1 snRNA in S-bodies as well, while condensed mRNAs was absent (Fig. 4D). These observations strongly suggested that S-bodies reflect condensates of stress-induced, mislocalized mature UsnRNPs in the cytoplasm rather than intermediates of possibly halted UsnRNP assembly. To verify this hypothesis, we blocked ongoing transcription or translation using NVP2 or Cycloheximide to stop ongoing UsnRNP biogenesis. Neither condition affected the condensation of hypermethylated U1snRNA or methylated Sm proteins in cytoplasmic S-bodies (Fig. S3).

Taken together, this confirms that osmotic stress transiently mislocalizes UsnRNPs from the nucleus to the cytoplasm, where they co-condense with the SMN complex.

### S-bodies reshape into Janus Bodies during stress recovery

Next, we tested whether the decondensation of S-bodies upon stress release may enable clearance of mislocalized UsnRNPs and whether residual cytoplasmic UsnRNPs may be associated with chronic stress.

We therefore investigated the phase of stress recovery in more detail. 20 min after release from osmotic stress, S-bodies were still visible even though they only appeared as droplet-like condensates (Fig. S4). Endogenous SMN visualized by indirect immunofluorescence remained in the condensates together with Gemin2 and DDX20 (Fig. S4A), methylated Sm proteins and hypermethylated CAP structures (Y12, TMG, Fig S4B).

**Fig. 5:**
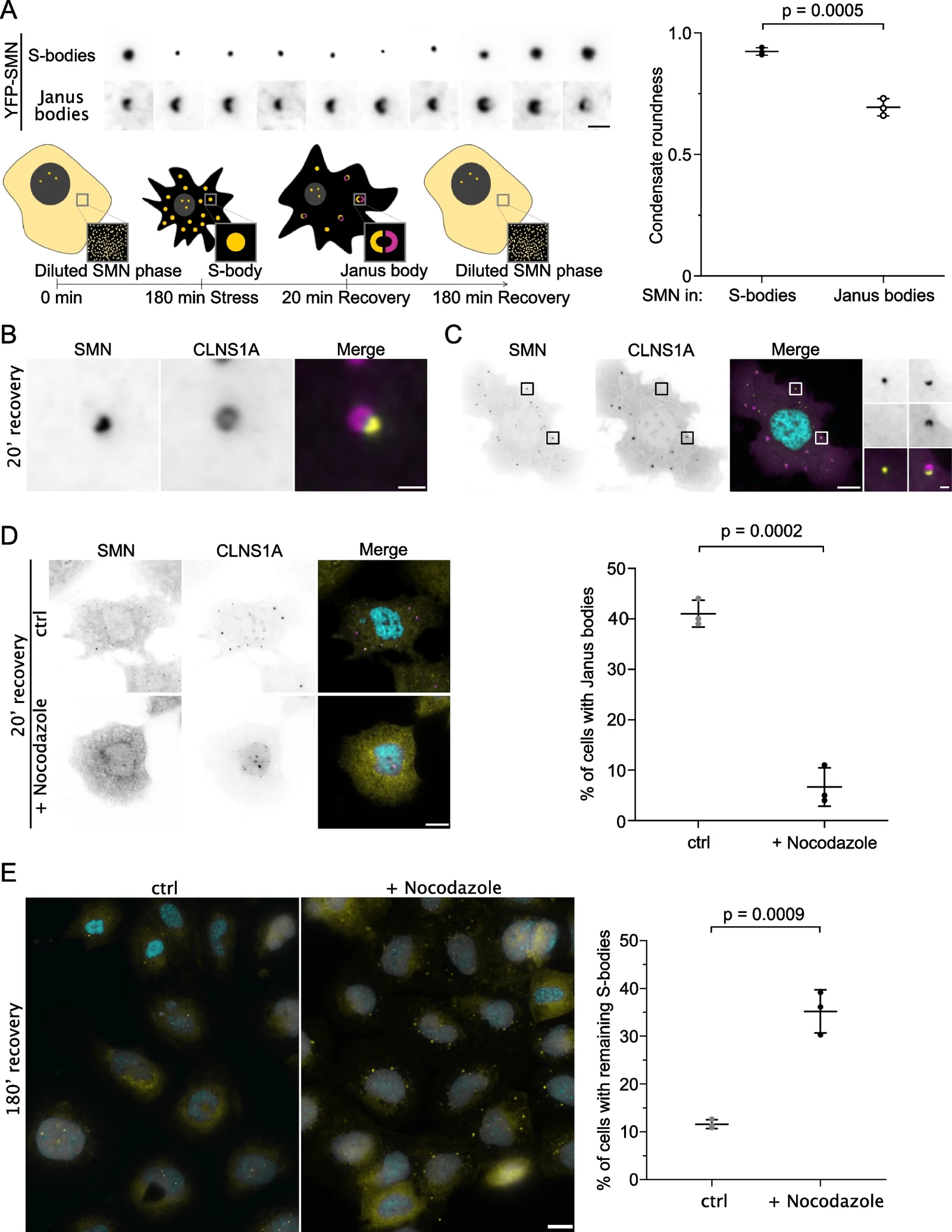
Recovery from osmotic stress reshapes S-bodies into biphasic Janus bodies. IMR90 cells were put under osmotic stress for 180 min and relieved from stress for further 20 min. SMN was visualized via indirect immunofluorescence. (**A**) Examples of shapes of the SMN signal at 180 min (S-bodies) and after additional 20 min of recovery (Janus bodies). Scalebar: 1 µm. Schematic representation of cytoplasmic SMN (yellow) and CLNS1A (magenta) condensates. The graph shows the shape analysis of SMN in S-bodies and Janus bodies. Condensate roundness measures how closely particles resemble a perfect circle (1.0) or elongated shapes (approaching 0.0). Plotted data points are mean values from 10 condensates of the same experiment while the mean from the three independent experiments is shown. -/+ S.D. and p-value is indicated. (**B**-**D**) SMN (yellow in merges) was analysed in parallel to CLNS1A/pICln. (**B**) Image detail to show two phases of Janus bodies with SMN and CLNS1A/pICln. Scalebar: 1 µm. (**C**) Single cell overview. Scalebar: 10 µm; in inset 1 µm. (**D**) Osmotic stress and recovery were compared with assembled MT (ctrl) or after MT disassembly upon nocodazole treatment 60’ prior to stress onset. Scalebar: 10 µm. Analysing 100 cells in each of 3 independent experiments revealed the fraction of cells with and without Janus bodies. Mean values and individual data points for each replicate are shown. -/+ S.D. and p-value is indicated. (**E**) Overview images of cells after 180’ recovery with assembled MT (ctrl) or after microtubule disassembly upon nocodazole treatment 60’ prior to stress onset. Scalebar: 10 µm. Analysing 100 cells in each of 3 independent experiments revealed the fraction of cells with remaining S-bodies. Mean values and individual data points for each replicate are shown. -/+ S.D. and p-value is indicated

At this time point of stress recovery, many S-bodies showed a hemisphere-like shape that was not observed before recovery onset. This difference in shape was consistent with SMN occupying only parts of a spherical condensate (Fig. 5A) with different components occupying the other phase. During UsnRNP assembly, the CLNS1A subunit of the PRMT5 complex interacts with Sm proteins and tightly cooperates with the SMN complex. Even though CLNS1A was not condensed into S-bodies under osmotic stress before recovery, we tested its association with S-bodies during the recovery phase. Indeed, indirect immunofluorescence detected CLNS1A condensates next to S-bodies whenever the geometry of the SMN signal indicated assembly of a second phase in the same droplet (Fig. 5B and Fig. S4C). In material sciences, droplets with separate but interconnected functions joint into a common condensate are termed “Janus” particles [34]. Accordingly, we named these biological two-phase condensates “Janus bodies” (JB). 20 min after release from osmotic stress, JB and spherical S-bodies only bearing SMN frequently coexisted in cells (Fig. 5C). U1 snRNA as well as the bulk of Sm proteins (detected by the Y12 antibody) remained at the SMN side of JB (Fig. S4B and S5A). Both JB and S-bodies largely dissolved after 180 min of stress recovery (Fig. S5B).

To address a potential role of MT binding for JB formation, we treated cells with the MT-depolymerizing drug Nocodazole prior to the release from osmotic stress. In the absence of MT, S-bodies were readily visible as droplet-like structures before (see Fig. 3C) and after stress recovery (Fig. 5D and E). However, we hardly observed any JB assembly, indicating that both needle-shaped S-bodies during stress and JB assembly during stress recovery require MT.

Taken together, our data demonstrate that S-bodies are rearranged in a MT-dependent manner during stress recovery and remain associated with cytoplasmic UsnRNPs until they dissolve. We also show a biological example of condensates with two separate phases of proteins, JB, suggesting that a physical and functional interconnection of the cytoplasmic UsnRNP assembly line comprising CLNS1A and SMN during osmotic stress. This interaction could shield UsnRNPs and subsequently allow efficient recovery from mislocalization and possibly misconformation after osmotic stress in the cytoplasm.

### S-bodies are functionally defective in SMA

Reduced activity of SMN strictly correlates with Spinal Muscular Atrophy (SMA) in humans. Defects of SMN’s function in UsnRNP biogenesis remain the best explanation for cell pathological phenotypes seen in SMA patients with loss-of-function mutation or deletion of *SMN1*.

**Fig. 6:**
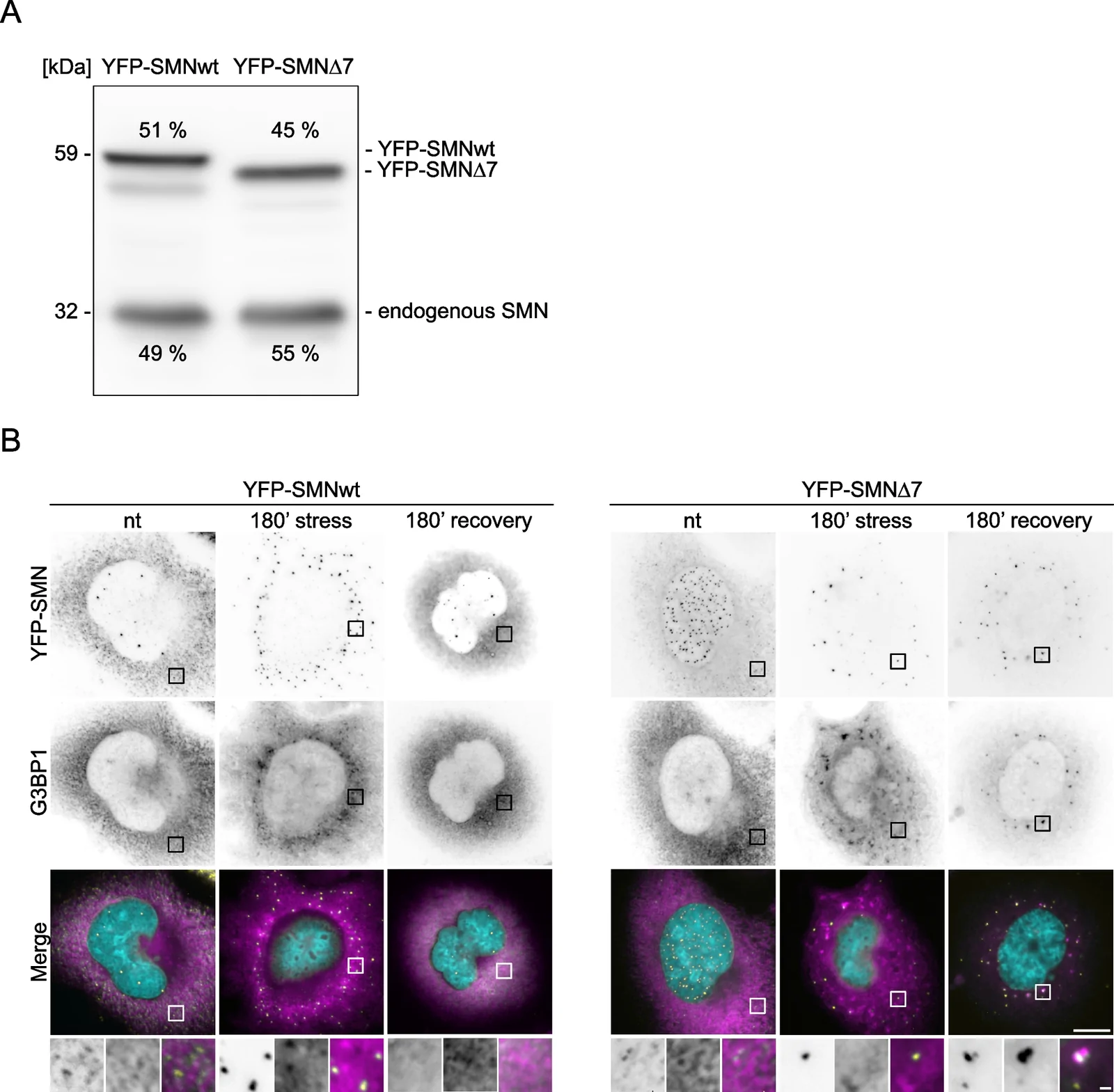
SMN full length (SMNwt) and SMN∆exon7 (SMN∆7) expressing cells differ in coping with osmotic stress. (**A**) Immunoblot to document the expression of exogenous (eYFP-SMNwt or eYFP-SMN∆7) and endogenous SMN in the IMR90 cell lines used here. (**B**) IMR90 eYFP-SMNwt or eYFP-SMN∆7 expressing cells were put under osmotic stress for 180 min and subsequently allowed to recover from stress for further 180 min. nt: non-treated, ie time 0’. YFP-SMN (yellow in merges) was detected by direct fluorescence in parallel to G3BP1 (magenta in merges) with indirect immunofluorescence. Scalebar: 10 µm; in inset 1 µm

To analyse if clearance of mislocalized UsnRNPs is affected in cellular SMA models, we used a human cell line expressing both endogenous full-length SMN1 comprising 294 amino acids and the pathologically shortened SMN variant that is largely produced from SMN2 transcripts (SMN missing exon 7: SMN*Δ*7) [26, 35, 36]. The overall SMN protein levels in eYFP-SMN*Δ*7 or eYFP-full-length SMN expressing cells were comparable (see Fig. 1 and following, Fig. 6A). However, S-body assembly in SMN*Δ*7 expressing cells was strongly reduced (Fig. 6B). Furthermore, the fewer S-bodies in SMN*Δ*7 cells forming upon osmotic stress, disassembled with slower kinetics, and showed increased association with the SG marker G3BP1 over time. This prolonged SG persistence suggests impaired stress recovery in cells expressing relatively high levels of the SMA-associated SMN variant (Fig. 6B), which possesses abnormal phase separation behaviour [27].

**Fig. 7:**
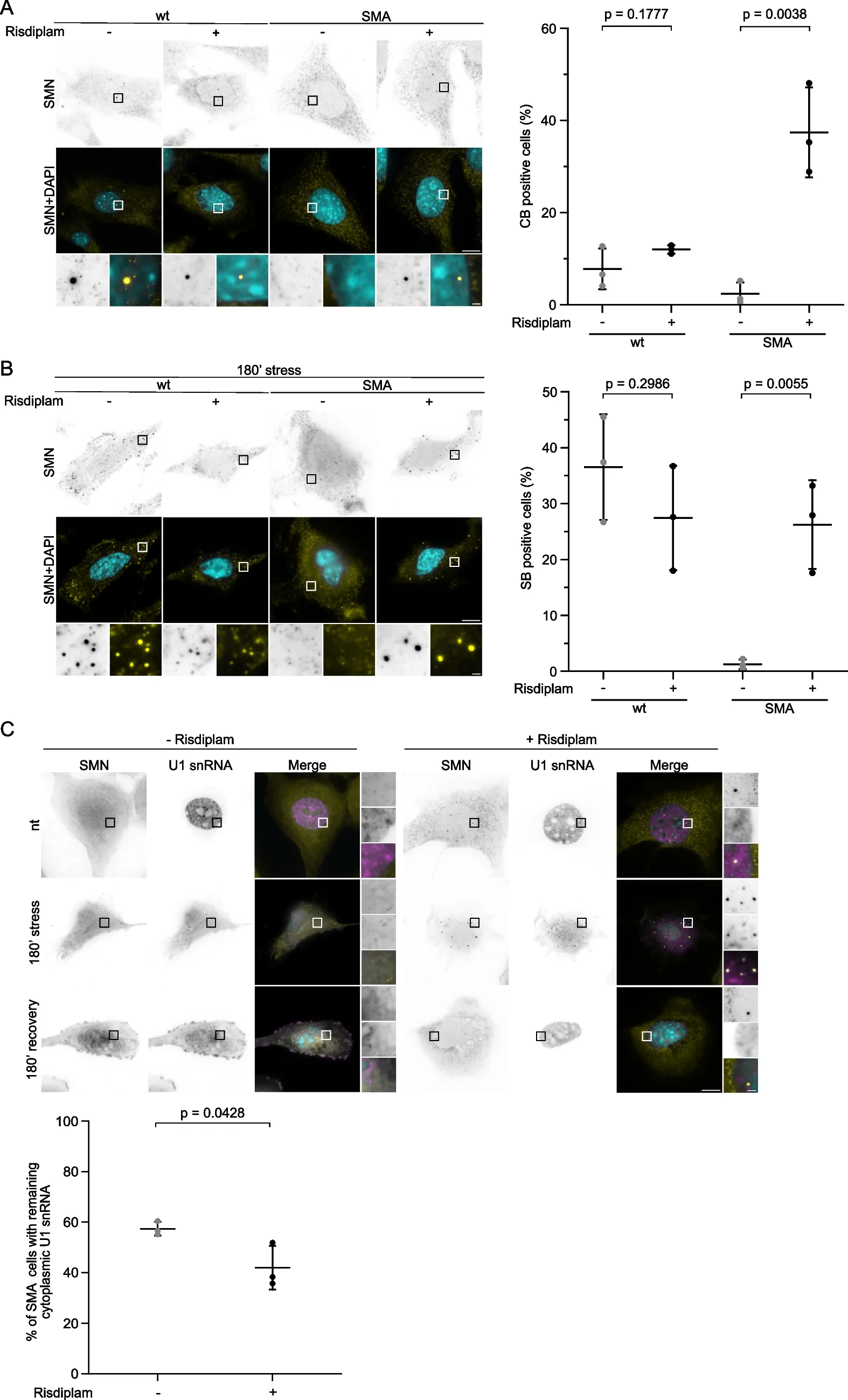
Osmotic stress and recovery in SMA model cells. Mouse embryonic fibroblasts (MEFs) expressing endogenous murine Smn (wt) or only human SMN2 from a single allele (SMA) were put under osmotic stress for 180’ without (-) or with (+) a 24 h Risdiplam treatment. SMN condensation in Cajal bodies (CB, A) or S-bodies (SB, B) was monitored by indirect immunofluorescence using an antibody that recognized identical epitopes in the murine or the human SMN protein. The graphs show quantifications of the cell fraction displaying CB (A) or SB (B). Plotted data points are mean values from 100 cells of the same experiment while the means from the three independent experiments are plotted. -/+ S.D. and *p*-values are indicated. (**C**) Cells as in (**A**) and (**B**) were allowed to recover for additional 180’ after osmotic stress and SMN (yellow in merges) analysed in parallel to U1 snRNA (magenta in merges) to monitor cytoplasmic UsnRNPs. The graph shows a quantification of SMA cells with remaining cytoplasmic U1 snRNA without (-) or with (+) Risdiplam treatment. Plotted data points are mean values from 50 cells of the same experiment while means from three independent experiments are shown. -/+ S.D. and p-value is indicated

To further analyse the significance of S-body function for SMA, we used mouse embryonic fibroblasts (MEFs) with partially humanized SMN.Mice only comprise one *Smn* gene, which simplifies knock-out. Introduction of human *SMN2* rescues lethality in knock-out cells albeit with lower levels of SMN (Fig. S6: 23% of wt Smn levels), recapitulating severe forms of SMA at the cellular level [37]. However, the *SMN2* splice site modifier Risdiplam restored expression levels back to 95% within 24 h (Fig. S6). Consistently, condensation of SMN visualized by indirect immunofluorescence of endogenous SMN in nuclear CBs or cytosolic S-bodies was markedly reduced in SMA-MEFs (Fig. 7A). In SMA MEFs treated with Risdiplam, CB condensation in non-stressed cells as well as S-body condensation upon osmotic stress, recovered [38] (Fig. 7A). Upon osmotic stress, wt and SMA MEFs displayed severe UsnRNP mislocalization (Fig. 7B). However, significantly fewer SMA cells than wt-MEFs could clear mislocalized UsnRNPs within 180 min from the cytoplasm (Fig. 7C).

Taken together, this demonstrates that pathologically reduced levels of full length SMN together with compromised phase separation of SMN*Δ*7 not only abolish SMN condensation but cause a significantly reduced capacity to cope with mislocalized UsnRNPs under osmotic stress. It further supports the idea that complete loss of SMN condensation in SMA patients and its associated reduced stress resilience is a driving force for systemic clinical symptoms in SMA accumulating over time and potentially explaining severity differences between different cell types.

## Discussion

### The significance of S-body assembly

The biophysical features of macromolecular entities enable them to condense by liquid–liquid phase separation (LLPS) and to switch between a diluted and a condensed state. The condensed, concentrated state not only scales up the local concentration of the entity but favors interactions with other biomolecules of the same condensate, while avoiding random interactions with any component of the same compartment not being concentrated in the condensate. The biochemical basis for LLPS founds itself in features that enable higher order assemblies such as intrinsically disordered domains and/or the polymeric nature of nucleic acids in ribonucleoprotein particles. The formation of biomolecular condensates has thus emerged as a general cellular principle to regulate the function of macromolecular complexes in general, and of RNPs in particular [39–43].

SMN had been shown to condense in CB before details of its function in sequential steps within the assembly line of UsnRNP biogenesis were elucidated [44]. However, colocalization with the protein constituents of UsnRNPs, i.e. Sm proteins, in CB further confirmed the hypothesis of SMN’s function in UsnRNP assembly [45, 46]. Ongoing UsnRNP assembly turned out to be the trigger for continuous condensation of SMN in CB [47]. In contrast, SMN remains not obviously condensed in the cytoplasm even though it governs the essential first steps in UsnRNP assembly. A potential role of cytoplasmic SMN condensation in UsnRNP biogenesis or homeostasis remained an obvious gap in understanding the molecular functions of SMN and its implications for SMA etiology.

In the experiments presented here, we show that osmotic compression introduces rapid but reversible condensation of cytoplasmic SMN complexes. S-bodies and granules positive for the SG marker G3BP1 do not colocalize at any point of osmotic stress or unperturbed stress recovery. This indicates that S-bodies represent a unique stress reactivity program specific to molecular crowding stress.

The condensation of cytoplasmic SMN in S-bodies is sensitive to DMSO. S-bodies, at least at stress onset, appear in droplets of homogenous size comparable to SMN condensation in CB. Both observations indicate that S-body droplets formed upon osmotic or mechanical stress occur via LLPS like SMN condensation in CB [28]. Osmotic compression has been assigned to cells under osmotic stress that not only occurs in plant cells but also as part of an integrated stress response in cells from yeast to man [31]. Likewise, mechanical stress results in the loss of solvent, increasing molecular crowding and resulting in the same fast condensation of cytoplasmic SMN. Conditions of mechanical stress feature not only tissues and organs under mechanical compression, but generic functional states such as mitosis, migration or travel through constrained passages when homing into a new niche [30].

### S-bodies in fire fighting mislocalized UsnRNPs

Our data indicate that these conditions, when tested experimentally mostly using osmotic stress, cause dramatic mislocalization of UsnRNPs from the nucleus to the cytoplasm. These, however, instantly condense together with SMN in the cytoplasmic condensates that we describe here as S-bodies. Several lines of evidence support the idea that UsnRNPs localized in S-bodies are mature UsnRNPs with a nuclear history. First, the signal of nuclear UsnRNPs decreases at the expense of an increase in cytoplasmic UsnRNP signal, while the nuclear signal increases again shortly after recovery. Second, modifications associated with nuclear import and nuclear residence of UsnRNPs are detected in S-bodies, including 5’CAP hypermethylation and Sm protein methylation. Third, pharmacological inhibition of both translation and transcription, blocking de novo UsnRNP synthesis, do not compromise S-body assembly and UsnRNP localization within. Assigning a main function of S-bodies to buffer misplaced UsnRNPs does not preclude that S-bodies also harbor assembly intermediates of ongoing UsnRNP biogenesis. Withdrawing ribosomal subunits from mRNAs via condensation in stress granules and notably P-bodies is a widely accepted principle of cellular regulation leading to a transient, reversible halt in translation [48]. Similarly, S-bodies may also work to safely stop UsnRNP production and to allow reinitiation of assembly after stress recovery.

How does the cell cope with a dramatic situation like this? First and foremost, cells recover quickly from osmotic pressure increase after stress relieve. Rapid condensation and decondensation of macromolecular entities have been described as a general, fast switch mechanism and reactivity of macromolecular complexes including RNPs. Macromolecular complexes can condense within seconds and decondense in a few minutes [31]. In contrast, the time for decondensation of YFP-SMN from S-bodies lies in the range of a couple of hours rather than minutes. This may suggest that S-body formation and disassembly is more complicated than other condensation-decondensation mechanisms previously described since it is coupled to other functions that become rate limiting for disassembly. Slow kinetics of disassembly are consistently explained with the scope of S-bodies towards UsnRNPs. To manage misplaced cytosolic UsnRNPs will require quality control and subsequent assignment of a final fate of cytoplasmic UsnRNPs. Nuclear retargeting is the most obvious destination. Consistently, the bipartite nuclear import signals, ie Sm core and hypermethylated CAP structure, could be detected as part of UsnRNPs in S-bodies. Different or additional import mechanisms of UsnRNPs may be required for recovery from osmotic stress. The increase in nuclear structures that comprise Sm methylation and 5’CAP hypermethylation during recovery observed here, however, underline that at least part of the mislocalized UsnRNPs are retargeted back to the nucleus.

As part of a cellular quality control mechanism, recovery may also involve the degradation of those UsnRNPs that are damaged irreversibly. Even though we do not have evidence for the latter pathway, SMN together with CLNS1A in a biological Janus body may provide an ideal microenvironment for safe UsnRNP disassembly and, potentially subsequent disassembly and degradation, consistent with previously described U1 snRNA degradation and UsnRNP surveillance mechanisms [49, 50]. Moreover, it has been demonstrated that high concentration of SMN and UsnRNPs together at the same time may allow the back-reaction of UsnRNP biogenesis [51]. CLNS1A and SMN act stepwise in the assembly, and they may chaperone the back reaction stepwise in the phases of JB.

### S-body dynamics alongside microtubules

While the initial condensation of S-bodies in droplets via LLPS is well comprehensible, further restructuring into needle-like condensates at later stages of stress, as well as formation of JB, are only to a much lesser extent. Needles and JB dramatically deviate from an energetically favored round droplet shape urging additional cellular activities that reshape S-bodies. We show that restructuring in both cases is MT dependent. Needle-like S-bodies likely elongate along MT as suggested by extended colocalization. It is well conceivable that the SMN complex combines features of a MT associated complex with biomolecular condensation under stress. This supposedly shifts SMN MT binding from sporadic, possibly weak monolayer binding to multilayered absorption and condensation along the MT surface [52]. Complete wetting along the MT surface [52] will energetically favor needle-like appearance of S-bodies. In contrast to the formation of needle-like S-bodies, restructuring into JB at the onset of recovery is not readily explained by a MT-dependent mechanism. Our microscopy data also document areas in JB that neither stain with SMN nor with CLNS1A antibodies, suggesting that these share JB with yet unknown components of a third phase. Further studies will be required to elucidate the organization and dynamics of JB.

### SMA model cells have lost S-body assembly and function

Binding to and traveling along MT is an attractive model to explain how cytoplasmic SMN complex functions are readily enabled even in the periphery of motor neurons. We show here that expression of the disease defining variant SMN*Δ*7 inhibits cytoplasmic condensation in recombinant cells and MEFs modelling the disease. Recently, SMN granules traveling in a MT dependent manner have been described in growth cones and neurites of cultured neurons. Overexpression of SMN*Δ*7 in these cells compromised cytoplasmic granule trafficking and neurite outgrowth [53]. Loss of the cytoplasmic condensation capacity and an associated loss in MT binding of SMN may thus contribute to the pathology of SMA beyond the primary function of SMN in ongoing UsnRNP assembly.

SMA cells lack the quality control pathway that monitors misplaced UsnRNPs and may, upon stress relieve, decide between nuclear retargeting and degradation of UsnRNPs. Which functions along these S-body-mediated UsnRNPs homeostasis steps are most affected in SMA cells is currently unclear, like long term consequences of misplaced UsnRNPs for disease etiology. Of note, S-body assembly and UsnRNP clearance could be rescued using the established SMA therapeutic Risdiplam for as little as 24 h. The fast recovery of S-body assembly and UsnRNP clearance within 24 h Risdiplam treatment is consistent with possible short-term effects of Risdiplam. Risdiplam will rescue protein doses of full length SMN. Our experiments in recombinant human IMR90 cells comparing exogenous expression of full length versus disease-defined SMN *Δ* 7 confirm that an intact C-terminus plays an important role in SMN condensation as indicated previously [26, 27].

Sensing and repair or degradation of cytoplasmic UsnRNPs ought to generally involve cellular stress signaling that detects the onset of mislocalization as well as the final clearance. Our data thus suggest that SMA patients may suffer from a general, non-tissue specific reduction in resilience against osmotic stress associated with deregulated stress reactivity. Experiments in fruit flies as model for SMA disease mechanisms revealed a dysregulation of innate immunity associated with perturbed UsnRNP biogenesis and pathologically reduced SMN function [54, 55]. SMA model mice regularly develop chronic inflammation at early stages of the disease [56] and SMA patient myoblast cells lines show signs of chronic activation of innate immunity signaling [57]. Consistently, chronic systemic inflammation beyond neural and muscle tissues observed in SMA patients has generated a new focus in current discussions about clinical symptoms of SMA [58]. This underlines that targeted modulation of innate immunity signals, e.g. by pharmacological inhibition of cGAS/STING signaling [59], arises as a promising strategy for rational refinement of current SMA therapies.

## Supplementary Information

Below is the link to the electronic supplementary material.

Supplementary file1 S-bodies exclude the stress granule marker G3BP1. Human IMR90 lung fibroblasts were left untreated (upper images) or put under stress for 180 minutes (DTT, Arsenite or sucrose for osmotic stress as indicated) and fixed with PFA. Indirect immunofluorescence detects endogenous SMN (yellow) and G3BP1 (magenta). Scalebar: 10 µm; in inset 1 µm (PDF 2361 KB)

Supplementary file2 S-bodies are sensitive to DMSO treatment. IMR90 cells were put under osmotic stress for 180 minutes and analysed by indirect immunofluorescence. During the last 10 minutes of stress, cells were treated without or with 10 % DMSO. Representative images are shown on left with SMN in yellow (scalebar: 10 µm; in inset 1 µm). The graph displays the categorization into S-body negative or positive cells without and with DMSO treatment. At least 100 cells were analysed in each of 3 independent experiments and mean values -/+ S.D. plotted (PDF 1547 KB)

Supplementary file3 S-body assembly is independent of ongoing UsnRNP biogenesis. IMR90 cells were put under osmotic stress for 180 minutes without or with cycloheximide treatment to block translation, or NVP2 treatment to inhibit transcription. Subsequent indirect immunofluorescence (A, C) or fluorescence in situ hybridization (B) visualises SMN (yellow in merges) and (A) methylated Sm protein (SYM, magenta in merges), (B) U1 snRNA (magenta in merges), or (C) the SmB protein (magenta in merges). Scalebar: 10 µm; in inset 1 µm (PDF 9488 KB)

Supplementary file4 SMN colocalizes to its interaction partners at the onset of stress recovery. IMR90 cells were put under osmotic stress for 180 minutes and relieved from stress for further 20 minutes. SMN (yellow in merges) and (A) Gemin2 or DDX20, or (B) methylated Sm proteins (Y12 antibody) and the hypermethylated UsnRNA CAP structures (TMG antibody) visualized by indirect immunofluorescence. Scalebar: 10 µm; in inset 1 µm. (C) Western blot showing a CLNS1A /pICln knockdown to confirm pICln antibody specificity (PDF 2877 KB)

Supplementary file5 U1snRNA colocalizes with SMN during stress recovery in JB. IMR90 cells were put under osmotic stress for 180’ and allowed to recover for 20’ or 180’ as indicated. Subsequent indirect immunofluorescence (B) and fluorescence in situ hybridization (A) visualises SMN (yellow in merges) and U1 snRNA (A), or CLNS1A (B). Scalebar: 10 µm; in insets 1 µm (PDF 1456 KB)

Supplementary file6 Risdiplam restores SMN expression levels in humanized Mouse Embryonic Fibroblasts. Mouse embryonic fibroblasts (MEFs) expressing endogenous murine Smn (wt) or human SMN from a single SMN2 allele (SMA) without (-) or with (+) a 24 h Risdiplam treatment were analysed for Smn/SMN expression by Western Blot. Please note that the antibody used here sees an identical epitope of the murine and the human protein recognizing both murine Smn and human SMN equally well. Tubulin was used as loading control and used to quantify the specific Smn/SMN signal (PDF 215 KB)

Supplementary file7 Time lapse imaging of live IMR90 cells stably expressing YFP-SMN, which were put under osmotic stress for 180 minutes and recovered under non-stressed conditions for another 180 minutes (MP4 147 KB)

Supplementary file8 Time lapse imaging in intervals of 10 s of live IMR90 cells stably expressing YFP-SMN, which were put under mechanical stress for 180 s (MP4 4820 KB)

## Data Availability

Data are available in the article itself and its supplementary materials.

## Abbreviations

CB: Cajal Body
Dd: Double distilled
DPBS: Dulbecco’s PBS
JB: Janus Body
LLPS: Liquid-liquid phase separation
MEF: Mouse Embryonic Fibroblast(s)
MT: Microtubule(s)
PHEM: Pipes Hepes EGTA MgSO_4_
P-body: Processing Body
S body: Cytoplasmic SMN condensate
SMA: Spinal muscular atrophy
SMN: Survival motor neurons
SMND7: SMN missing exon 7
SG: Stress granules
STORM: Stochastical Optical Reconstruction
TMG: 2,2,7 Trimethylguanosine
U body: Uridin rich body
