# Supplementary material for: Biphasic SMN condensates safeguard stress-induced cytoplasmic mislocalization of splicing RNPs
Source: Cell Mol Life Sci. 2026 Sep 30;83(1):350. doi: 10.1007/s00018-026-06459-9 (PMC13627572; doi:10.1007/s00018-026-06459-9)

Riedel_Dressler_FigureS4

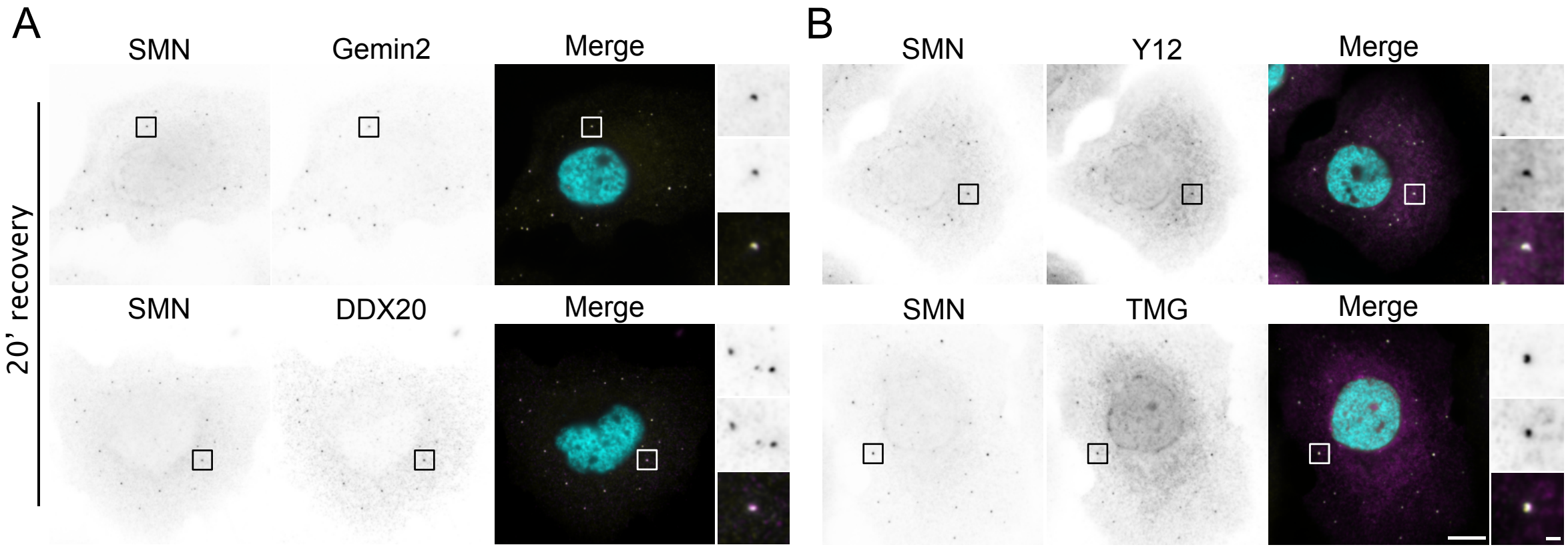

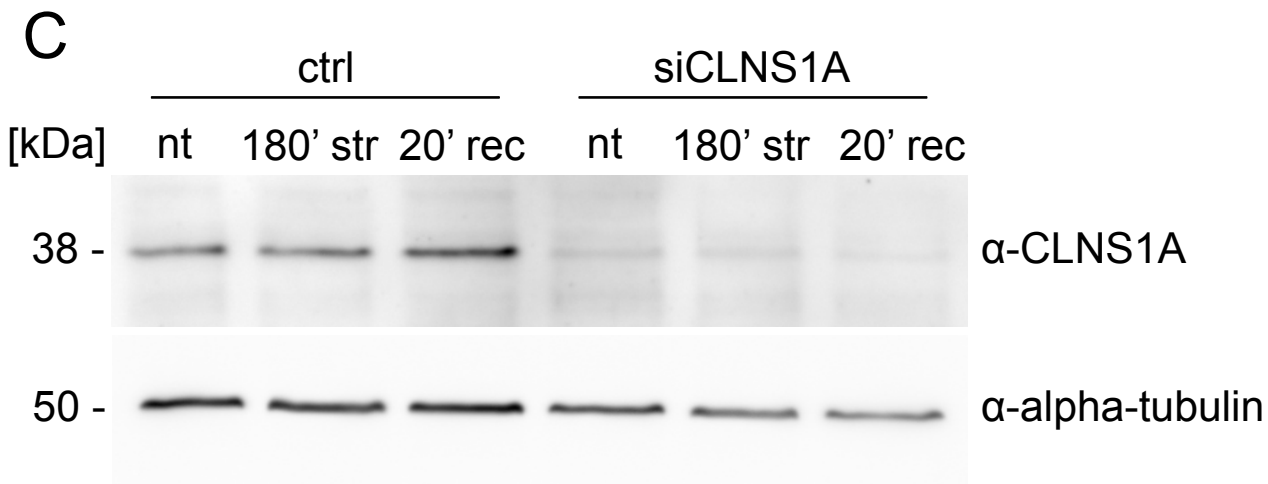

Supplement: Supplementary file 4 [file 18_2026_6459_MOESM4_ESM.pdf]
